# Molecular characterization of cathepsin B from *Clonorchis sinensis *excretory/secretory products and assessment of its potential for serodiagnosis of clonorchiasis

**DOI:** 10.1186/1756-3305-4-149

**Published:** 2011-07-27

**Authors:** Wenjun Chen, Xiaoyun Wang, Xuerong Li, Xiaoli Lv, Chenhui Zhou, Chuanhuan Deng, Huali Lei, Jingtao Men, Yongxiu Fan, Chi Liang, Xinbing Yu

**Affiliations:** 1 Department of Parasitology, Zhongshan School of Medicine, Sun Yat-sen University, Guangzhou 510080, People's Republic of China; 2 Key Laboratory for Tropical Diseases Control, Sun Yat-sen University, Ministry of Education, Guangzhou 510080, People's Republic of China; 3 Department of Gynecology and Pediatrics, Nursing School, Guangdong Medical College, Dongguan, 523808, PR China

## Abstract

**Background:**

Cathepsin cysteine proteases play multiple roles in the life cycle of parasites such as food uptake, immune invasion and pathogenesis, making them valuable targets for diagnostic assays, vaccines and drugs. The purpose of this study was to identify a cathepsin B of *Clonorchis sinensis *(*Cs*CB) and to investigate its diagnostic value for human helminthiases.

**Results:**

The predicted amino acid sequence of the cathepsin B of *C. sinensis *shared 63%, 52%, 50% identity with that of *Schistosoma japonicum*, *Homo sapiens *and *Fasciola hepatica*, respectively. Sequence encoding proenzyme of *Cs*CB was overexpressed in *Escherichia coli*. Reverse transcription PCR experiments revealed that *Cs*CB transcribed in both adult worm and metacercaria of *C. sinensis*. *Cs*CB was identified as a *C. sinensis *excretory/secretory product by immunoblot assay, which was consistent with immunohistochemical localization showing that *Cs*CB was especially expressed in the intestine of *C. sinensis *adults. Both ELISA and western blotting analysis showed recombinant *Cs*CB could react with human sera from clonorchiasis and other helminthiases.

**Conclusions:**

Our findings revealed that secreted CsCB may play an important role in the biology of C. sinensis and could be a diagnostic candidate for helminthiases.

## Background

*Clonorchis sinensis *is the causative agent of clonorchiasis, a chronic liver infection of human acquired through consumption of raw or undercooked fish and shrimps with infectious metacercariae. Clonorchiasis is endemic in Asian countries and over 35 million people globally are infected *C. sinensis*, including an estimated 15 million in People's Republic of China [1]. Recently, this infection has emerged in non-endemic regions and developed countries following growing international markets, improved transportation systems and demographic changes such as population movements [2]. *C. sinensis *adults reside chronically in the biliary tract and cause periductal inflammation, fibrosis, pyogenic cholangitis, biliary calculi, cholecystitis, liver cirrhosis and pancreatitis [3]. Like *Opisthorchis viverrini*, *C. sinensis *is one of the direct causes of cholangiocarcinoma announced by the International Agency for Research on Cancer (IARC) in 2009 [4]. It is important to take some measures to control clonorchiasis due to its public health threat. Until now, the main prevention and control strategies for this parasite are treatment of individual patients with praziquantel, and interrupting transmission at the intermediate host level [5]. However, there have been little effective measures to prevent this neglected tropical disease [6].

Cysteine proteinase is ubiquitous in all species [7-9]. In parasites, cysteine proteases have attracted much attention for their essential roles in parasite physiology as well as in host-parasite interactions through their modulation of various pathobiological events, including host tissue invasion, nutrient uptake, host immune evasion and molting [10-13]. Research has been conducted to characterize the biochemical properties and pathophysiological roles of cysteine proteases from trematode parasites. The essential roles of cysteine proteases in parasite survival or growth make them attractive targets for vaccines or chemotherapeutic agents [14-16]. Several genes encoding *C. sinensis *cysteine proteases have been identified and partially characterized [17-19]. Lee et al. [20] reported that cathepsin F-like cysteine protease of *C. sinensis *is a good vaccine candidate against clonorchiasis. Li et al. [21] found that endogenous cysteine proteases of *C. sinensis *metacercariae are probably involved in the excystment process. Kang et al. [22] indicated that partially purified cysteine protease from excretory/secretory products (ESP) of *C. sinensis *adults exhibited significant cytotoxic effects against cultured cells. ESP of parasites have attracted more attention for their significant roles in the diagnosis, vaccine, drug target and host-parasite interactions etc. *In vitro *biochemical studies have predicted that ESP from liver flukes have definitive roles in feeding behavior, detoxification of bile components and immune evasion [23]. Ju et al. [18] have identified legumain from ESP as a serodiagnostic antigen of clonorchiasis. In addition, several genes encoding *C. sinensis *cysteine proteases have also been identified and their value as diagnostic antigens for clonorchiasis was investigated [24,25]. However, little is known about cathepsin B (CB) in *C. sinensis *except five distinct sequences deposited in Genbank.

As members of the cysteine protease family, cathepsins have been assayed in the serodiagnosis of both human and animal in parasite infections. Cornelissen et al. [26] reported a specificity of 75.3% in naturally infected cattle using *Fasciola hepatica *cathepsin L as coating antigen. Carnevale et al. [27] found that recombinant pro-cathepsin-L was 100% specific in the diagnosis of human *F. hepatica *infection. Sripa et al. [28] indicated that *Ov*-CB-1 was acceptable in ELISA for the serodiagnosis of human opisthorchiasis with 67% and 81% of sensitivity and specificity, respectively. To find out whether CB could be applied for serodiagnosis in *C. sinensis *infection, we identified a gene encoding cathepsin B of *C. sinensis *(*Cs*CB) and investigated its diagnostic value for human helminthiases.

## Results

### Sequence analysis of *Cs*CB gene sequence

**Figure 1:**
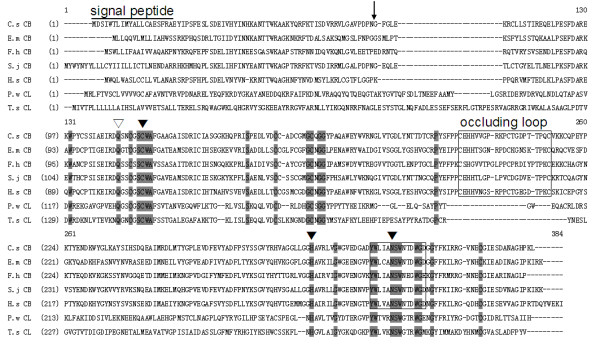
**Alignment of cathepsin B (*Cs*CB) deduced amino acid sequence from *C. sinensis *and other species**. The deduced amino acid sequence shares 63%, 52%, 50% and 53% identity with CB of *S. japonicum*, *H. sapiens*, *F. hepatica *and *E. multilocularis*, respectively. Highly conserved residues are shaded in *gray*. Residues of the catalytic triad (Cys_116_, His_285 _and Asn_305_) are indicated with *black triangles*. The oxyanion Gln is marked with *white triangle*; *arrow *shows cleavage point of mature enzyme, the occluding residues and hemoglobinase motif are *boxed*.

The complete coding sequence of *Cs*CB is comprised of 1,020 bp encoding a putative protein of 339 amino acids with a predicted molecular mass of 37.9 kDa and an isoelectric point of 5.32. The ORF consists of a hydrophobic signal peptide at the N-terminus, followed by a pro-region of between 70 and 71 AA and a mature protease sequence. BLASTx showed that it shared 63%, 52%, 50% and 53% identity with CB of *Schistosoma japonicum*, *Homo sapiens*, *F. hepatica *and *Echinococcus multilocularis*, respectively. Highly conserved residues of the catalytic triad (Cys_116_, His_285 _and Asn_305_) as well as the Gln could be searched in this sequence. The putative protein contained an occluding loop that is the signature of cathepsin Bs and a haemoglobinase motif which is shared by helminth blood-feeders (Figure 1).

### Cloning, expression and purification of recombinant *Cs*CB (r*Cs*CB) in *E. coli*

**Figure 2:**
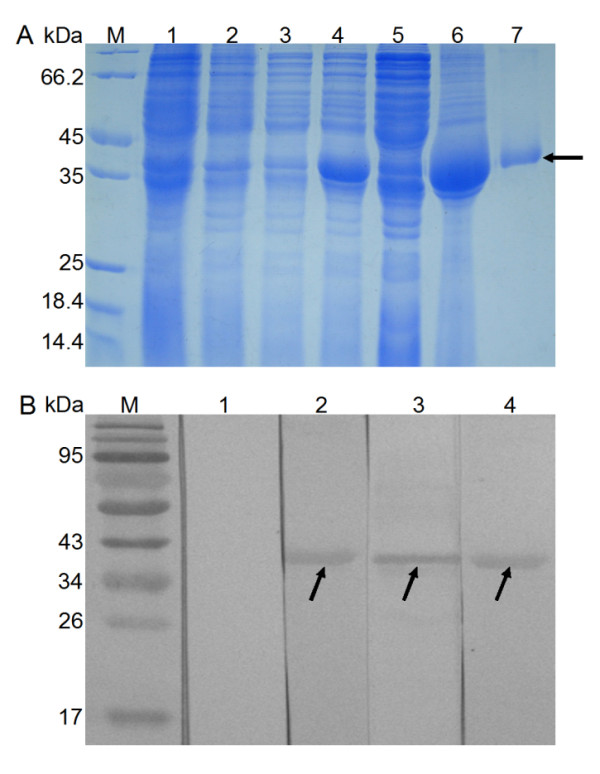
**Expression, purification and identification of *Cs*CB**. A. Expression and purification of *Cs*CB by 12% SDS-PAGE. M, protein molecular weight markers; lane 1, lysate of *E. coli *with pET-28a(+) without IPTG induction; lane 2, lysate of *E. coli *with pET-28a(+) with IPTG induction, lane 3, lysate of *E. coli *with pET28a(+)-*Cs*CB before induction; lane 4, lysate of *E. coli *with pET28a(+)-*Cs*CB after induction; lane 5, supernatant of induced pET28a(+)-*Cs*CB; lane 6, sediment of induced pET28a(+)-*Cs*CB; lane 7, purified *Cs*CB. B. Western blotting analysis of recombinant *Cs*CB. Lane 1, recombinant *Cs*CB reacted with the sera from normal rats; lane 2, recombinant *CsCB *reacted with the sera from rats immunized with recombinant *Cs*CB; lane 3, recombinant *Cs*CB reacted with the sera from *C. sinensis-*infected rats; lane 4, recombinant *Cs*CB reacted with the sera from rats immunized with the ESP.

The recombinant pET-28a(+) plasmid containing the CB gene coding region (signal peptide removed) was confirmed by digestion with restriction enzyme. DNA sequencing revealed that the construct was correct with 6 × His tag at the N terminus of the recombinant protein. The recombinant *Cs*CB was expressed in inclusion bodies in *E. coli*. SDS-PAGE showed that the molecular mass of fusion protein was about 40 kDa, which was in correspondence with the predicted 39.2 kDa (including 34 amino acids of vectors). After purification and renaturation, the concentration of the recombinant protein was about 0.25 mg/ml (Figure 2A).

### Identification of *Cs*CB as ESP by western blotting

Rat anti-r*Cs*CB antibody titers were higher than 1:102,400 as determined by ELISA. In western blotting assay, both *C. sinensis-*infected rat serum and anti-*Cs*CB rat serum could react with the *Cs*CB while the normal rat serum could not. Comparing with the control group, sera from rats immunized with total ESP could recognize *Cs*CB. In addition, ESP has been shown to react with anti-*Cs*CB rat serum (data not shown). The results above indicated that *Cs*CB was a component of ESP (Figure 2B).

### RT-PCR analysis of *Cs*CB at life-stage of *C.sinensis*

**Figure 3:**
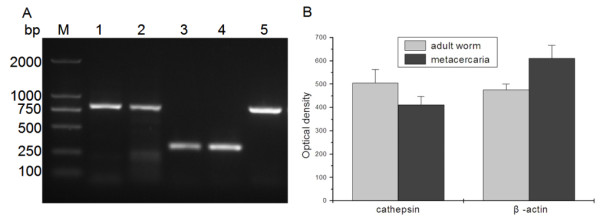
**Transcriptional level of *C. sinensis *CB at metacercaria and adult worm**. A**. **1% agarose gel. DNA marker (M), *Cs*CB PCR products amplified from adult worm cDNA (lane 1), metacercaria cDNA (lane 2), β-actin amplified from adult worm cDNA (lane 3), metacercaria cDNA (lane 4) and *Cs*CB PCR product amplified from recombinant CB plasmid (lane 5). B. PCR products were quantified and analyzed. PCR products were quantified by Tanon Gis software, compared normalized by *C. sinensis *β-actin, analyzed by Student's *t *test (Software package SPSS16.0). *P*-value of < 0.05 indicated statistical significance.

*Cs*CB transcrips were detected both in adults and metacercaria of *C. sinensis *(Figure 3A), and the expression level in adults was higher than that of metacercaria when normalized by β-actin (Figure 3B, P < 0.05).

### Immunohistochemical localization of CsCB in *C. sinensis *adults

**Figure 4:**
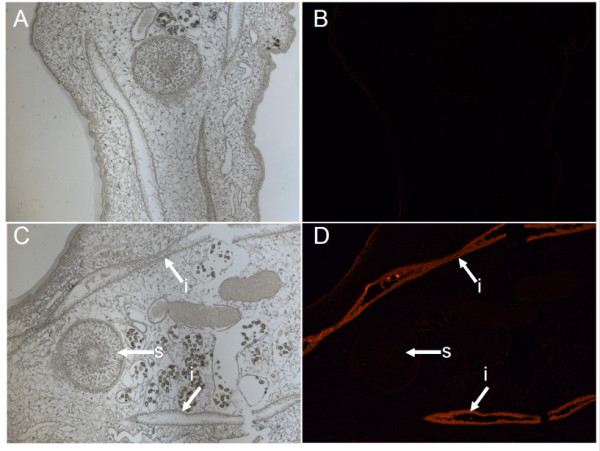
**Immunolocalization of *Cs*CB in adult worm of *C. sinensis***. Rat anti-*Cs*CB serum was used as primary antibody and goat anti-rat IgG as the secondary antibody. Panel A and D show tissues of adult worm under fluorescence microscope. Panel A and C show the same part under white light. Panel A and B were the negative control treated with preimmune rat serum. i, intestine s, sucker. The images were magnified at 100× for photograph.

Using the antisera against *Cs*CB as the primary antibody and fluorescence labeling IgG as the secondary antibody, immunolocalization showed that *Cs*CB distributed in the intestine of adults, while no specific fluorescence except autofluorescence was detected in adults treated with normal serum (Figure 4).

### Serodiagnosis of human helminthiases

**Figure 5:**
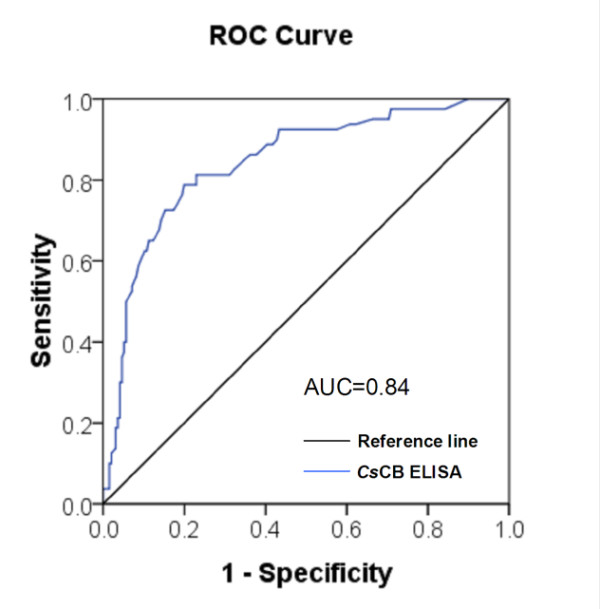
**ROC analysis of performance of r*Cs*CB as a serodiagnostic antigen**. ROC curve of r*Cs*CB employed as the antigen in ELISA, calculated from OD_450 _values of sera from parasitologically positive and negative clonorchiasis individuals. The curve was plotted between sensitivity and 1-specificity for different cut-off values of OD_450_. The curve showed that the optimal cut-off value at OD_450 _was 0.47 with the area under curve (AUC) was 0.84.

**Figure 6:**
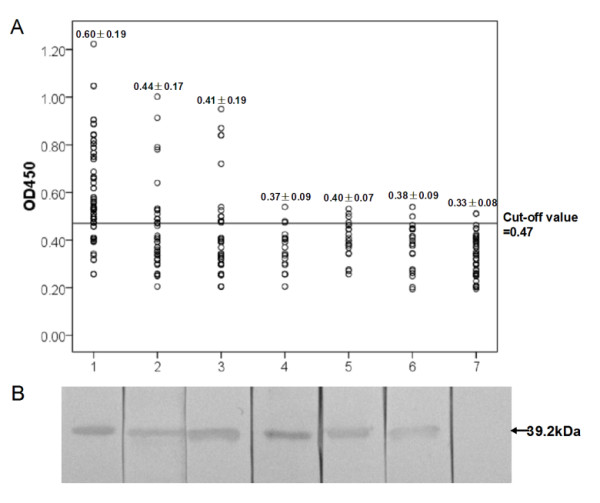
**Immune response to human helminthiases of r*Cs*CB**. 1-7: clonorchiasis, fascioliasis, schistosomiasis, paragonimiasis, echinococciosis, cysticercosis and healthy serum, respectively. A**. **Serodiagnosis of human helminthiases by ELISA(scatter plot). B**. **Immune reactionsshown by western blotting. The probed bands were indicated with arrow.

The optimal concentration of coating antigen and dilution of serum samples determined by checker board titration were 3 μg/ml and 1:400, respectively. The cut-off value for positive infection status in ELISA tests evaluated by receiver operating characteristic (ROC) curve was 0.47 (Figure 5). Sera from humans infected with *C. sinensis *showed a sensitivity of 79% by ELSA based on r*Cs*CB and healthy sera and sera from those infected with other helminths showed a specificity of 81% (Table 1). Both ELISA and western blotting assays showed that r*Cs*CB could react with human sera from clonorchiasis, fascioliasis, schistosomiasis, paragonimiasis, cysticercosis and echinococciosis (Figure 6).

**Table 1:** Summary of difference between r*Cs*CB ELISA and stool examinations results.

Panel A			
**CsCB ELISA**	**No. of samples from stool examination**		
	
	**Negative**	**Positive**	**Total**

Negative	158(81%)	17	175
Positive	38	63(79%)	101
Total	196	80	276

**Panel B**			

Parasites	No. of serum samples		No. positive of *Cs*CB ELISA

*Echinococcus multilocularis*	22		3(14%)
*Fasciola hepatica*	42		15(36%)
*Schistosoma japonicum*	41		13(32%)
*Paragonimus westermani*	21		4(19%)
*Taenia solium*	20		2(10%)
negative	50		1(2%)
Total	196		38(19%)

**Panel A: **Comparison of the r*Cs*CB ELISA with microscopic examination of stool samples as the gold standard method for diagnosis of clonorchiasis in 276 human sera. r*Cs*CB ELISA test showed 81% specificity and 79% sensitivity with reference to stool examination. **Panel B: **Number of the sera from individuals infected with other helminths used for cross-reactivity tests.

## Discussion

Cathepsins in general are of interest to parasitologists, as there is considerable evidence that they play a key role in the biology of parasites [29]. In this study, a CB of *C. sinensis *was cloned and overexpressed in *E. coli*. It was classified as CB due to its sequence homology to cathepsin B protein and structure. The putative amino acid sequence shared 63%, 52% and 50% identities with cathepsin B from *S. japonicum*, *H. sapiens *and *F. hepatica*, respectively. Sequence analysis showed that *Cs*CB has typical catalytic residue of cysteine, histidine and asparagine, as well an occluding loop that is the signature of cathepsin Bs [30]. A haemoglobinase motif which is shared by helminth blood-feeders could be found in this deduced sequence [31]. Since *C. sinensis *generally feed on bile and epithelial cells rather than blood, however, it is thought that this motif may be an important tool for identifying potential hemoglobinases and contribute to haemoglobin degradation [32]. The occluding loop is a distinctive feature of CBs; it is not only responsible for exopeptidase activity to CBs, but also governing the pH dependence of auto-activation. In *F. hepatica*, Beckham [33] reported that recombinant FhCatB1 did not auto-activate upon secretion by yeast but could be auto-activated in a low pH buffer. As it stands, *Cs*CB may develop auto-activation in an appropriate pH buffer, so further studies will be carried out to investigate the auto-activation and enzyme activity of this protease.

Cathepsin B proteases of parasitic helminths such as *F. hepatica *and *Schistosoma mansoni *have been well studied. In *F. hepatica*, for example, there are at least 10 genes encoding CB being identified by using proteomics and transcriptome analysis, among which six are components of ESP of immature flukes. RNAi directed to *Fh*CB2 reduced invasion of *F. hepatica *newly excysted juveniles through the intestine *in vitro *and vaccine with *F. hepatica *CB2 reduced fluke burdens and liver damage in rats following challenge, revealing that *Fh*CB2 was a key molecule in *F. hepatica *biology and drug therapy [34]. In *S. mansoni*, *Sm*CB1 and *Sm*CB2 have been identified and the function of *Sm*CB1 was examined using RNAi silencing, which illustrated that *Sm*CB1 played a role in nutrient acquisition [35,36]. Based on the key role of CB in the biology of parasites, we expect that *Cs*CB may play the same role in nutrition intake and immune invasion of host. In multicellular parasites such as trematodes, the intestine is a major source of secreted proteases and also a place for nutrition digestion and absorption. According to results of immunolocalization, *Cs*CB was distributed in the intestine of adult worm, indicating that *Cs*CB might be involved in digestion of host protein and nutrient uptake for this parasite itself. In our RT-PCR experiments, *Cs*CB could be detected in both adult worm and metacercaria of *C. sinensis*. As a secreted protease, these observations suggested that *Cs*CB may play an important role in the biology of this parasite.

Proteases contained in ESP of parasites released to the environment, play key roles in bile duct malignancy and the subsequent development of cholangiocarcinoma [37,38]. Our previous studies revealed that ESP of adult *C. sinensis *lysophospholipase and phospholipase A2 might be pathogenic factors of human hepatic fibrosis caused by infection of *C. sinensis *[39,40], which could deepen our understanding of the pathogenesis of *C. sinensis*. CB has already been found in ESP of *C. sinensis *and some other helminths [41]. Moreover, western blotting revealed that *Cs*CB was a component of ESP, and *Cs*CB could react with *C. sinensis*-infected rat serum. In ELISA results, rats injected with recombinant *Cs*CB developed high antibody titers. Our results showed that *Cs*CB might be involved in the pathogenesis of *C. sinensis*-related hepatobiliary diseases as a component of ESP with antigenicity and immunogenicity. Indeed, *C. sinensis *adults dwell in the bile duct of host, persistently released ESP resulting in mechanical damage and chemical stimulus which must have brought on infiltration of inflammatory cells surrounding bile ducts and adenomatous hyperplasia of biliary epithelia [42-44]. However, there remains much to do to elucidate the mechanism of *C. sinensis *causing hepatobiliary diseases. We have also approached the antigenicity and diagnostic value of *Cs*CB in human helminthiases. Both the ELISA and western blotting showed that *Cs*CB could react with sera from most of human helminthiases, but not with sera from healthy people. High identity in the predicted amino acid sequences of *C. sinensis *and other helminths could be responsible for the observed cross-reactivity. Though cross-reactivity existed, *Cs*CB could also be applied as a diagnostic candidate of clonorchiasis since treatment with praziquantel has the same efficacy on helminths.

## Conclusion

A gene encoding cathepsin B protein of adult *C. sinensis *was cloned and expressed for the first time. Our findings revealed that *Cs*CB, which is expressed in the intestine and released outside the worm as a component of ESP, may play an important role in the biology of *C. sinensis*. Moreover, CB may play conserved roles in helminths for the high homology analyzed by bioinformatics. Further investigations are required to characterize the conserved functions of such important protease and the role as a potential vaccine candidate against *C. sinensis *infection.

## Materials and methods

### Sequence analysis of *Cs*CB gene sequence

A complete coding sequence encoding CB was isolated from GenBank (Accession no. EF102086.1) in NCBI http://www.ncbi.nlm.nih.gov/, the physicochemical properties and the functional domains in deduced amino acids were predicted by proteomics tools in ExPaSy web site http://www.expasy.org/. Based on the similarity, the homologous sequences of different species, including *E. multilocularis *(BAJ83491.1), *F. hepatica*(ABU62925.1), *S. japonicum *(CAX71086.1), *H. sapiens *(NP_001899.1), *Paragonimus westermani *(AAB93494.1) and *Taenia solium *(AAS00027.1) were identified using the basic local alignment search tool (BLAST) server. Multiple sequences alignments were performed using bioinformatics analysis software Vector NTI suite 8.0.

### Cloning, expression, purification and refolding of *Cs*CB in *E. coli*

Gene sequence encoding *Cs*CB (signal peptide excluded) was amplified from cDNA of *C. sinensis *by polymerase chain reaction (PCR) using forward primer: 5'-CGCGGATCCGAGTATATTCCATCTTTCGA-3' and the reverse primer: 5'-GTCCTCGAGTCACAGTTTTGGATGACC-3' with *BamH*I/*Xho*I restriction enzyme sites (underlined). PCR was carried out for 30 cycles at 94°C for 45 s, 57°C for 45 s, and 72°C for 60 s, and the reaction continued for 10 min at 72°C after the last cycle. Purified PCR products were cloned into the His_6 _tag expression vector pET-28a(+) (Novagen; USA) with corresponding incision enzymes. The recombinant plasmid was transformed into *E. coli *for expression and insertion confirmed by digestion with restriction enzyme and DNA sequencing. Expression of recombinant *Cs*CB protein was induced by isopropyl-β-D-thiogalactoside (IPTG) at a final concentration of 0.2 mM for 3 h at 30°C. The bacterial cells were collected by centrifugation at 4°C, and the inclusion bodies containing the recombinant fusion protein were solubilized completely with 6 M urea in 20 mM Tris-HCl buffer (pH 8.0), followed by purification with His Bind Purification kit (Novagen; USA) and elution with 150 mM imidazole. Renaturation was carried out by stepwise diluting urea in dialysate buffer (20 mM Tris-HCl, 5 mM EDTA buffer, pH 8.0). Purified protein was analyzed by sodium dodecyl sulfate-polyacrylamide gel electrophoresis (SDS-PAGE) and stained with Coomassie blue, final recombinant protein concentration was estimated by Bradford assay using BSA method used as a standard.

### Semi-quantitative reverse transcription-PCR (RT-PCR) analysis of *Cs*CB

In order to determine the mRNA transcriptional level of *Cs*CB in various stages of the parasite, total RNA of adult worms and metacercariae were extracted and quantitated by nucleic acid/protein analyzer (Beckman Coulter; USA). Reverse transcription reactions were carried out by transforming equal amounts of total RNA (1 μg each) to cDNA using RT-PCR Kit (TaKaRa; PR China). RT-PCR experiments were employed to amplify the transcripts of *Cs*CB from cDNA of adult worms and metacercariae, respectively, and *C. sinensis *β-actin (GenBank accession no. EU109284) was used as a positive control. The forward and reverse primer for *Cs*CB were 5'-GGATTCGGCCTGGAAAAAC-3', 5'-CAGTTTTGGATGACCAGCAT-3' and for β-actin were 5'-GGTGACGCTGAAGTATCCTATTGA-3', 5'-CCAAAGCATAGCCCTCGTAGAT-3', respectively. The programs for PCR were the same as described above. PCR products from two life stages were quantified by Tanon Gis software (Tanon 4100; PR China) normalized by *C. sinensis *β-actin and analyzed by Student's *t *test (Software package SPSS16.0), *P*-value of < 0.05 indicated statistical significance.

### Preparation for the antisera of recombinant protein and *C. sinensis *excretory/secretory product (ESP)

Living adults of *C. sinensis *were collected and cultured in RPMI-1640 (Gibco; USA) at 37°C under 5% CO_2 _for 6 h. The culture was centrifuged at 12,000× g at 4°C for 30 min to remove insoluble debris. The supernatant was dialyzed in PBS for 12 h and concentrated with sucrose. Six-week-old male Sprague-Dawley rats were purchased for animal experiments under the Guide for the Care and Use of Laboratory Animals. Both the recombinant CB and ESP were emulsified with complete Freund's adjuvant and immunized subcutaneously to SD rats. Each rat was given 200 μg r*Cs*CB or ESP at the first injection, and 100 μg protein (emulsified with incomplete Freund's adjuvant) was given for 2 booster injections at 2-week intervals; antisera were collected before each injection and serum antibody responses to r*Cs*CB and ESP were tested by ELISA.

### SDS-PAGE and western blotting

The recombinant protein (1 μg per lane) was subjected to SDS-PAGE and electrotransferred onto polyvinylidene difluoride membrane (PVDF, Whatman; USA) at 100 v for 1 h. The membrane was blocked with 5% skim milk in phosphate buffered saline tween-20 (PBST, pH 7.4) at 37°C for 2 h, washed 5 times with PBST, then incubated with different antisera (antisera against the recombinant *Cs*CB protein, antisera against the ESP, sera from *C. sinensis*-infected rats and normal rats, respectively, 1:200 dilutions for all sera) at 37°C for 2 h. After washing, the membrane was incubated with rabbit anti-rat IgG HRP-conjugated horse radish peroxidase (1:2,000 dilution; Boster; PR China) at 37°C for 1 h. Diaminobenzidine (DAB) substrate solution (Invitrogen, USA) was used to visualize the reaction according to the manufacturer's instructions.

### Immunolocalization of *Cs*CB protein in *C. sinensis *adult worm

*C. sinensis *adult worms fixed with 10% neutral formalin were embedded with paraffin and sliced into 3-5 μm thickness. After deparaffinating with dimethyl benzene and gradient alcohol, all sections were blocked with normal goat serum at 4°C overnight, then incubated with rat anti-*Cs*CB serum for 2 h at room temperature (RT), the sera from normal rats were used as negative control, the serum was diluted at 1: 200 for all sections. The sections were subsequently incubated with goat anti-rat IgG labeled with red-fluorescent Cy3 (1: 400 dilutions with 0.1% BSA in PBS, Jackson, USA) for 1 h at RT in dark and imaged using fluorescent microscope (ZEISS; Germany).

### Source of serum samples

A total of 276 human serum samples were employed in order to approach the antigenicity and diagnostic value of *Cs*CB in human helminthiases. 80 sera from clonorchiasis patients were collected from endemic areas in Guangxi Province and were egg-positive proved by microscopic examination of stool samples. Other human sera including single infection of *E. multilocularis *(22), *F. hepatica *(42), *S. japonicum *(41), *P. westermani *(21), *T. solium *(20) and 50 samples of serum from healthy humans were provided and diagnosed by Centers for Disease Control and Prevention of Nanning, Jiangsu, Gansu and Fujian provinces. Sera were retrieved and stored in aliquots at -20°C until use.

### Serodiagnosis of human helminthiases by ELISA and western blotting

The optimal antigen concentration and serum dilution required for ELISA were determined by checker board titration. Briefly, 96-well microtiter plates (Costar, USA) were coated with 100 μl 1 μg/ml, 3 μg/ml, 5 μg/ml, 7 μg/ml r*Cs*CB (in 0.05 mol/l NaHCO3 buffer, pH 9.6) and incubated at 4°C overnight. After three washings with PBST, the microplates were blocked with 5% skimmed milk (*w*/*v*, in PBST containing 0.1% BSA) for 2 h at 37°C. Following another washing procedure, the plates were incubated with human helminthiases sera (1:50, 1:100, 1:200, 1:400 dilutions for each coating concentration in PBST containing 0.1% BSA) for 2 h at 37°C. Goat anti-human IgG (1:10,000 dilutions in 0.1% BSA-PBST recommended by producer; Invitrogen, USA) were employed as the secondary antibody. Subsequent reactions were developed with 3', 3', 5', 5'-tetramethyl benzidine (TMB; BD Biosciences; USA) and stopped with 2 M H_2_SO4. All assays were tested in triplicate and repeated twice. The absorbance value was measured at 450 nm.

In western blotting assay, r*Cs*CB was subjected to 12% SDS-PAGE and electrotransferred onto PVDF membrane (Whatman; USA). The membrane was incubated with different human helminthiases sera (clonorchiasis, echinococciosis, fascioliasis, schistosomiasis, paragonimiasis, and cysticercosis, respectively) which showed high OD_450 _values by ELISA test at 37°C for 2 h, human healthy serum was used as negative control, all sera were 1:400 diluted in PBST containing 0.1% BSA. Goat anti-human IgG HRP-conjugated horse radish peroxidase (1:2,000 dilution; Boster; PR China) was the secondary antibody to react with the membrane and DAB substrate solution was used to visualize the reaction.

### Statistical analysis

Software package SPSS16.0 was used in the present study for all statistical analysis. Briefly, Student's *t *test was used to analyze the measurement data among groups. *P *value of < 0.05 was considered statistically significant. The optimal cut-off value was calculated based on ROC curve analysis which correlated with sensitivity and 1-specificity [45]. ROC curve, area under the curve (AUC) and scatter plot were carried out using SPSS16.0. The sensitivity and specificity were calculated using microscopic examination of stool samples as gold standard method.

### Ethical approval

Centers for Disease Control and Prevention of Nanning, Jiangsu, Gansu and Fujian provinces granted ethical approval for human sera used in the present study. All animal experiments were approved by institute's ethical committee of Sun Yat-sen University.

## Competing interests

The authors declare that they have no competing interests.

## Authors' contributions

WJC, XYW and XBY conceived and designed the experiments; WJC, XYW, XRL, CHZ, XLL, CHD, HLL, JTM, YXF and CL performed the experiments; WJC, XYW, XRL and XLL analyzed the data. All authors read and approved the final manuscript.
